# Whole Genomic Analysis of Human G12P[6] and G12P[8] Rotavirus Strains that Have Emerged in Myanmar

**DOI:** 10.1371/journal.pone.0124965

**Published:** 2015-05-04

**Authors:** Tomihiko Ide, Satoshi Komoto, Kyoko Higo-Moriguchi, Khaing Win Htun, Yi Yi Myint, Theingi Win Myat, Kyaw Zin Thant, Hlaing Myat Thu, Mo Mo Win, Htun Naing Oo, Than Htut, Mitsutaka Wakuda, Francis Ekow Dennis, Kei Haga, Yoshiki Fujii, Kazuhiko Katayama, Shofiqur Rahman, Sa Van Nguyen, Kouji Umeda, Keiji Oguma, Takao Tsuji, Koki Taniguchi

**Affiliations:** 1 Department of Virology and Parasitology, Fujita Health University School of Medicine, Toyoake, Aichi, Japan; 2 Nay Pyi Taw General Hospital (Central Myanmar), Nay Pyi Taw, Myanmar; 3 Department of Medical Research (Upper Myanmar), Pyin Oo Lwin, Myanmar; 4 Department of Medical Research (Lower Myanmar), Yangon, Myanmar; 5 Department of Traditional Medicine (Central Myanmar), Nay Pyi Taw, Myanmar; 6 Ministry of Health (Central Myanmar), Nay Pyi Taw, Myanmar; 7 Department of Virology II, National Institute of Infectious Diseases, Musashi-Murayama, Tokyo, Japan; 8 Department of Electron Microscopy and Histopathology, Noguchi Memorial Institute for Medical Research, College of Health Sciences, University of Ghana, Legon, Ghana; 9 Immunology Research Institute in Gifu, EW Nutrition Japan, Gifu, Japan; 10 Department of Bacteriology, Okayama University Graduate School of Medicine, Dentistry, and Pharmaceutical Sciences, Okayama, Japan; 11 Department of Microbiology, Fujita Health University School of Medicine, Toyoake, Aichi, Japan; Tulane University, UNITED STATES

## Abstract

G12 rotaviruses are emerging rotavirus strains causing severe diarrhea in infants and young children worldwide. However, the whole genomes of only a few G12 strains have been fully sequenced and analyzed. In this study, we sequenced and characterized the complete genomes of six G12 strains (RVA/Human-tc/MMR/A14/2011/G12P[8], RVA/Human-tc/MMR/A23/2011/G12P[6], RVA/Human-tc/MMR/A25/2011/G12P[8], RVA/Human-tc/MMR/P02/2011/G12P[8], RVA/Human-tc/MMR/P39/2011/G12P[8], and RVA/Human-tc/MMR/P43/2011/G12P[8]) detected in six stool samples from children with acute gastroenteritis in Myanmar. On whole genomic analysis, all six Myanmarese G12 strains were found to have a Wa-like genetic backbone: G12-P[8]-I1-R1-C1-M1-A1-N1-T1-E1-H1 for strains A14, A25, P02, P39, and P43, and G12-P[6]-I1-R1-C1-M1-A1-N1-T1-E1-H1 for strain A23. Phylogenetic analysis showed that most genes of the six strains examined in this study were genetically related to globally circulating human G1, G3, G9, and G12 strains. Of note is that the NSP4 gene of strain A23 exhibited the closest relationship with the cognate genes of human-like bovine strains as well as human strains, suggesting the occurrence of reassortment between human and bovine strains. Furthermore, strains A14, A25, P02, P39, and P43 were very closely related to one another in all the 11 gene segments, indicating derivation of the five strains from a common origin. On the other hand, strain A23 consistently formed distinct clusters as to all the 11 gene segments, indicating a distinct origin of strain A23 from that of strains A14, A25, P02, P39, and P43. To our knowledge, this is the first report on whole genome-based characterization of G12 strains that have emerged in Myanmar. Our observations will provide important insights into the evolutionary dynamics of spreading G12 rotaviruses in Asia.

## Introduction

Group A rotavirus (RVA), a member of the family *Reoviridae*, is the major cause of severe dehydrating diarrhea in the young of humans and many animal species worldwide. In humans, RVA infections are associated with high morbidity and mortality, being responsible for an estimated annual 453,000 deaths among children <5 years of age [1]. More than half of these deaths were estimated to occur in developing countries in Asia and Africa [1, 2]. In Myanmar, a high disease burden of RVA infection in children (<5 years old) was found in studies conducted in Yangon, RVAs being identified in 53–57% of fecal samples from diarrheal disease cases in the years 2002–2005 [3, 4].

The RVA virion is a triple-layered, non-enveloped icosahedron that encloses an 11-segment genome of double-stranded (ds)RNA [5]. RVA has two outer capsid proteins, VP7 and VP4, which are implicated independently in neutralization, and define the G and P genotypes, respectively. To date, RVAs have been classified into at least 27 G and 37 P genotypes [6, 7]. Among them, 5 G (G1-4 and G9) and 3 P (P[4], P[6], and P[8]) genotypes are commonly associated with human infections [8, 9]. Over the last decade, the global emergence of unusual G12 strains has been a matter of concern, and G12 seems to be the sixth major human G genotype [9–11].

The first G12 strain, L26 (G12P[4]), was detected in children <2 years old with acute diarrhea in the Philippines in 1987 [12, 13]. More than 10 years later, G12 strains began to emerge globally, predominantly in combination with either a P[6] or P[8] genotype and less commonly with a P[4] or P[9] genotype [9–11, 14–17]. In Asia, G12 strains are being increasingly identified in diarrheic children in several countries; Bangladesh [10, 18–22], Bhutan [23], India [24–33], Indonesia [34], Japan [16], Kazakhstan [35], Korea [36], Kyrgyzstan [35], Nepal [37–40], Pakistan [41, 42], Sri Lanka [43], Thailand [14, 15, 44–46], and Vietnam [47], indicating the continued spread of G12 strains in Asia. In 2011, we detected 29 G12 strains from diarrheic children in Central Myanmar enrolling a total of 54 RVA-positive stool specimens by PCR-based G and P genotyping (Ide et al., in preparation).

A whole genome-based genotyping system was recently proposed for RVAs based on assignment to all the 11 gene segments (i.e., G/P and non-G/P genes) [48]. In the new genotyping system, the acronym Gx-P[x]-Ix-Rx-Cx-Mx-Ax-Nx-Tx-Ex-Hx, where x is an integer, defines the genotype of the VP7-VP4-VP6-VP1-VP2-VP3-NSP1-NSP2-NSP3-NSP4-NSP5 genes of a given RVA strain. The Wa-like strains are characterized by non-G/P genotypes (I1-R1-C1-M1-A1-N1-T1-E1-H1), and tend to have G/P genotypes G1P[8], G3P[8], G4P[8], and G9P[8] [49]. In contrast, the DS-1-like strains are characterized by non-G/P genotypes (I2-R2-C2-M2-A2-N2-T2-E2-H2), and tend to have G/P genotype G2P[4]. The third minor AU-1-like strains are characterized by non-G/P genotypes (I3-R3-C3-M3-A3-N3-T3-E3-H3), and tend to have G/P genotype G3P[9]. Whole genome-based analysis is a reliable method for obtaining conclusive data on the origin of an RVA strain, and for tracing its evolutionary pattern [48, 50]. To date, the whole genome sequences of only a few G12 strains have been fully sequenced and characterized, which provided evidence that the recently detected Asian G12 strains with the Wa-like genotype backbone are distantly related to prototype Asian G12 strains L26 (G12P[4]) and T152 (G12P[9]) [14] having the DS-1-like and AU-1-like genotype backbone, respectively [10]. However, as the overall genomic constellation and the exact evolutionary pattern of Asian G12 strains remain to be elucidated, whole genomic analysis of Myanmarese G12 strains might be useful for obtaining a more precise understanding of the evolutionary pattern of emerging G12 strains in Asia. In the present study, we analyzed the whole genomes of six G12 strains that have emerged in Myanmar. Moreover, in this study, deep sequencing with the next generation sequencing (NGS) Illumina MiSeq platform was carried out to obtain the complete nucleotide sequences of the whole genomes of these six G12 strains.

## Materials and Methods

### Ethics statement

The study was approved by the Ethical Committee on Medical Research Involving Human Subjects of the Department of Medical Research (Central Myanmar). In this study, written informed consent for the testing of stool samples for RVAs and characterization of identified RVA strains was obtained from children’s parents/guardians.

### Virus strains

The full-genomic sequences were determined for strains A14, A23, A25, P02, P39, and P43, which were identified as the pathogens causing diarrhea in six stool specimens from children with acute diarrhea during the RVA strain surveillance in the Pediatric Infectious Disease Wards of the Defense Services Obstetrics, Gyneacology and Children’s Hospital, Central Myanmar in 2011, which involved a total of 54 RVA-positive fecal samples (Ide et al., in preparation). Stool samples containing strains A14, A23, A25, P02, P39, and P43 were kept at −30°C until use.

### Virus isolation

Stool samples suspended in PBS containing 0.5 mM MgCl_2_ and 1 mM CaCl_2_ were inoculated onto monkey kidney cell line MA104 for virus isolation [51], and the cultures were serially passaged two more times in MA104 cells. The viral dsRNAs were extracted from the cell cultures using a QIAamp Viral RNA Mini Kit (Qiagen). The extracted dsRNAs were used for (i) polyacrylamide gel electrophoresis (PAGE) analysis, and (ii) whole genomic analysis. For PAGE analysis, the dsRNAs were electrophoresed in a 10% polyacrylamide gel for 16 h at 20 mA at room temperature, followed by silver staining [52] to determine the genomic dsRNA profiles. For whole genomic analysis, viral dsRNAs were subjected to Illumina MiSeq sequencing as described below.

### cDNA library building and Illumina MiSeq sequencing

Preparation of a cDNA library and Illumina MiSeq sequencing were performed as reported previously [49, 53]. Briefly, a 200 bp fragment library ligated with bar-coded adapters was constructed for individual strains using an NEBNext Ultra RNA library Prep Kit for Illumina v1.2 (New England Biolabs) according to the manufacturer’s instructions. Library purification was performed using Agencourt AMPure XP magnetic beads (Beckman Coulter). The quality of the purified cDNA libraries was assessed on a MultiNA MCE-202 bioanalyzer (Shimadzu). Nucleotide sequencing was performed on an Illumina MiSeq sequencer (Illumina) using a MiSeq Reagent Kit v2 (Illumina) to generate 151 paired-end reads. Data analysis was carried out using CLC Genomics Workbench v7.0.3 (CLC Bio). Contigs were assembled from the obtained sequence reads by *de novo* assembly. Using the assembled contigs as query sequences, the Basic Local Alignment Search Tool (BLAST) non-redundant nucleotide database was searched to obtain the full-length nucleotide sequence of each gene segment of the six strains. To fill in missing sequence gaps for the NSP2 gene of strain A23, and the NSP5 genes of strains A14 and A25, specific primers were used to process viral dsRNAs of the three strains using Sanger sequencing [54]. The primer pairs used for cDNA amplification of the NSP2 and NSP5 genes are (+) 5’-GGCTTTTAAAGCGTCTCAGTC-3’ and (-) 5’-GGTCACATAAGCGCTTTCTATTC-3’, and (+) 5’-GGCTTTTAAAGCGCTACAGTG-3’ and (-) 5’-GGTCACAAAACGGGAGTGGGG-3’, respectively [54]. The final full-length genomes for the six strains were assembled using a combination of Illumina MiSeq and Sanger sequencing data. The nucleotide sequences were translated into amino acid sequences using GENETYX v11 (GENETYX).

### SNP detection

Single nucleotide polymorphisms (SNPs) were called using CLC Genomics Workbench v7.0.3 using all available sequencing data with at least 100 sequence reads covering each nucleotide position. A variant frequency threshold of ≥1% per site was used and approximate variant P-values were calculated.

### Determination of RVA genotypes

The genotype of each of the 11 gene segments of the six strains was determined using the RotaC v2.0 automated genotyping tool (http://rotac.regatools.be/) [55] according to the guidelines proposed by the Rotavirus Classification Working Group (RCWG).

### Phylogenetic analyses

Sequence comparisons were carried out as described previously [54]. Briefly, multiple alignment of each viral gene was performed using CLUSTAL W [56]. Phylogenetic trees were constructed using the maximum likelihood method and the Kimura 2-parameter substitution model using MEGA6.06 [57]. The reliability of the branching order was estimated from 1000 bootstrap replicates [58]. The results of phylogenetic analyses were validated using several other genetic distance models, such as the Jukes-Cantor, Tamura 3-parameter, Hasegawa-Kishino-Yano, and Tamuta-Nei ones (data not shown).

### Nucleotide sequence accession numbers

The nucleotide sequence data presented in this paper have been deposited in the DDBJ and EMBL/GenBank data libraries. The accession numbers for the nucleotide sequences of the VP1-4, VP6-7, and NSP1-5 genes of strains A14, A23, A25, P02, P39, and P43 are LC019041-LC019051, LC019052-LC019062, LC019063-LC019073, LC019074-LC019084, LC019085-LC019095, and LC019096-LC019106, respectively.

## Results and Discussion

### Isolation of strains A14, A23, A25, P02, P39, and P43 in cell culture

**Fig 1:**
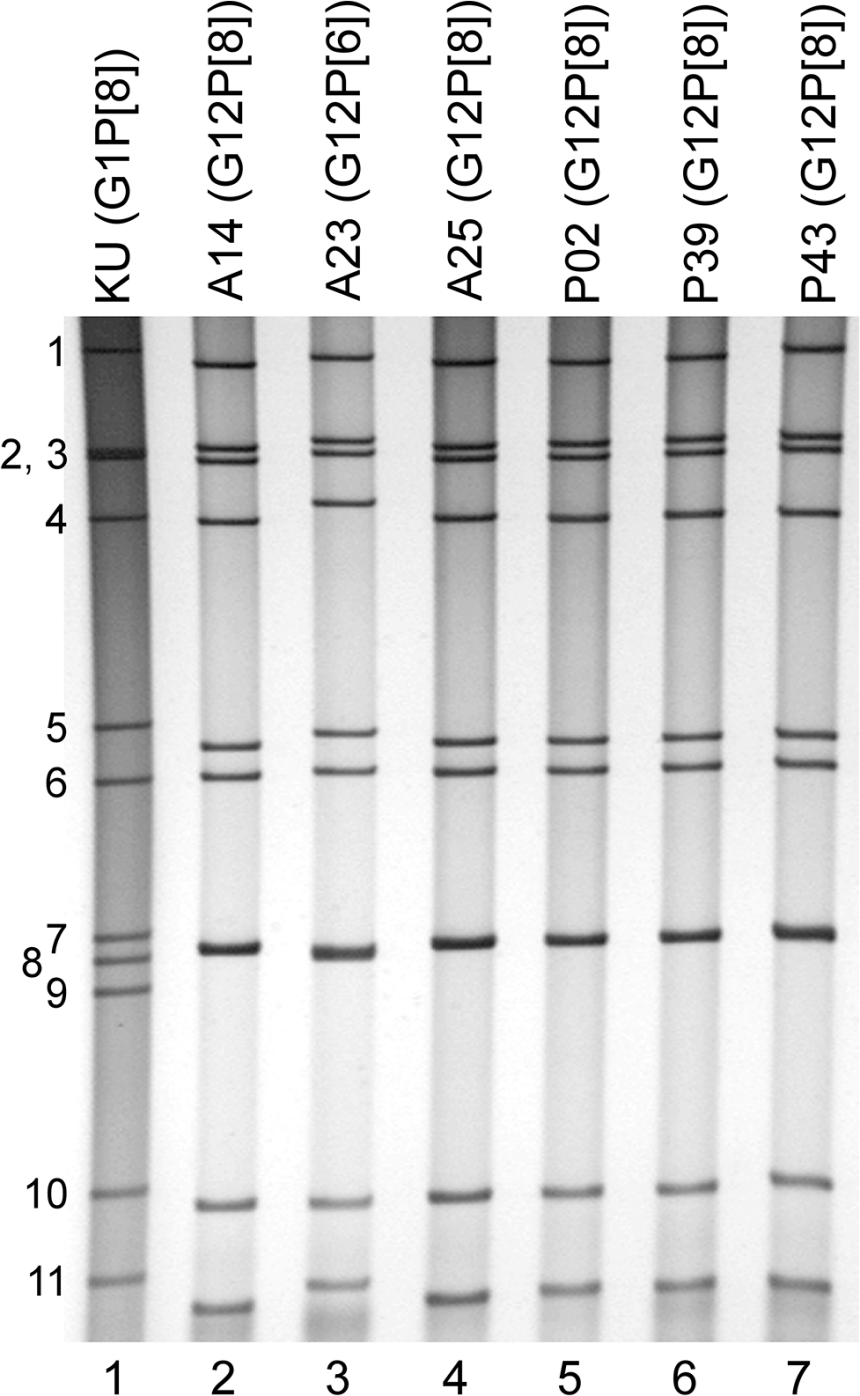
Genomic dsRNA profiles of strains A14, A23, A25, P02, P39, and P43. Lane 1, dsRNAs of strain KU (G1P[8]) extracted from a cell culture; lanes 2–7, dsRNAs of strains A14 (lane 2), A23 (lane 3), A25 (lane 4), P02 (lane 5), P39 (lane 6), and P43 (lane 7) extracted from cell cultures. The numbers on the left indicate the order of the genomic dsRNA segments of strain KU.

To obtain enough genomic material for whole genome sequencing of the emerging G12 strains in Myanmar, we primarily attempted to isolate strains A14, A23, A25, P02, P39, and P43 using the MA104 cell line; all six strains could be cell culture-adapted. Virion dsRNAs were extracted and then analyzed by PAGE. Fig 1 shows the profiles of the viral dsRNAs from human strain KU (G1P[8]), as a reference (lane 1), and strains A14 (lane 2), A23 (lane 3), A25 (lane 4), P02 (lane 5), P39 (lane 6), and P43 (lane 7) from the cell cultures. The individual dsRNA migration pattern in PAGE of cell culture-adapted strains A14, A23, A25, P02, P39, and P43 was identical to that of the original strains A14, A23, A25, P02, P39, and P43 present in stool samples, respectively (data not shown). They all showed a long electropherotype. Cell culture-adapted strains A14, A23, A25, P02, P39, and P43 were named RVA/Human-tc/MMR/A14/2011/G12P[8], RVA/Human-tc/MMR/A23/2011/G12P[6], RVA/Human-tc/MMR/A25/2011/G12P[8], RVA/Human-tc/MMR/P02/2011/G12P[8], RVA/Human-tc/MMR/P39/2011/G12P[8], and RVA/Human-tc/MMR/P43/2011/G12P[8], respectively, according to the guidelines for the uniformity of RVAs proposed by the RCWG. Of note was that strains A14, A25, P02, P39, and P43 showed an almost identical electropherotype, suggesting a close genetic relatedness among the five strains.

### Nucleotide sequencing and whole-genome-based genotyping of strains A14, A23, A25, P02, P39, and P43

In order to gain an insight into the genetic variability among strains A14, A23, A25, P02, P39, and P43, and the genetic relatedness with other RVA strains worldwide, the full-genome sequences of all the 11 segments of these six strains were determined using the NGS Illumina MiSeq platform. The whole genomes of the six strains were amplified using a sequence-independent primer set and then sequenced successfully. Illumina MiSeq sequencing yielded 2.0 x 10^6^ reads (~143 bp average length), 1.9 x 10^6^ reads (~144 bp average length), 1.7 x 10^6^ reads (~144 bp average length), 1.8 x 10^6^ reads (~145 bp average length), 1.7 x 10^6^ reads (~142 bp average length), and 1.7 x 10^6^ reads (~145 bp average length) for strains A14, A23, A25, P02, P39, and P43, respectively. Missing sequence information in the contigs of the NSP2 gene of strain A23, and the NSP5 genes of strains A14 and A25 was filled in by Sanger sequencing of RT-PCR products. This approach allowed determination of the complete or nearly complete nucleotide sequences of all the 11 segments of the six strains, while deep sequencing revealed the presence of multiple SNPs at <1.8% frequency in the six strains.

**Fig 2:**
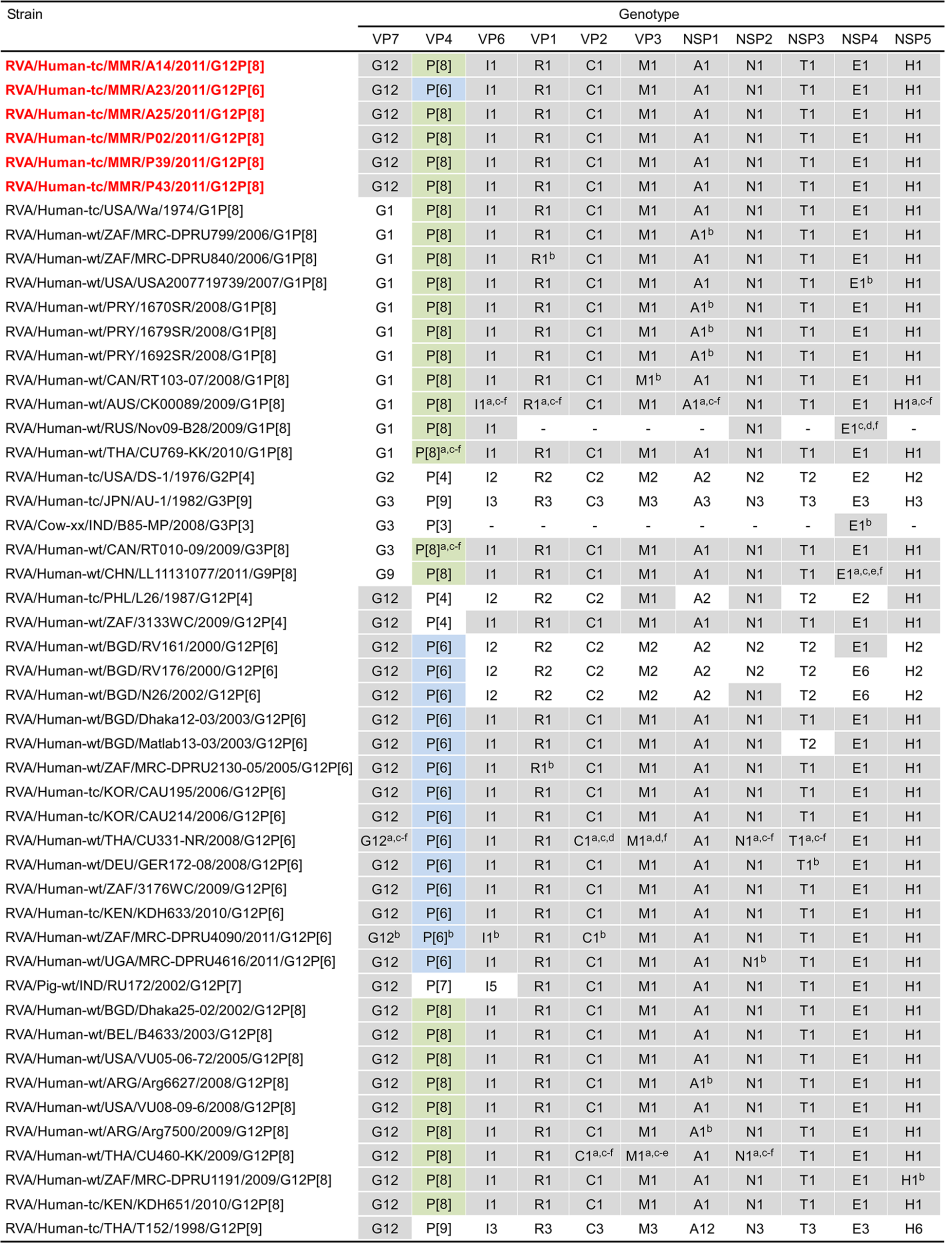
Genotype natures of the 11 gene segments of six Myanmarese G12 strains, A14, A23, A25, P02, P39, and P43, with those of selected human and animal strains. Strains A14, A23, A25, P02, P39, and P43 are shown in red. Gray shading indicates the 10 gene segments (VP7, VP6, VP1-3, and NSP1-5) with genotypes identical to those of strains A14, A23, A25, P02, P39, and P43. Green shading indicates the VP4 gene segments with a P[8] genotype identical to those of strains A14, A25, P02, P39, and P43. Blue shading indicates the VP4 gene segments with a P[6] genotype identical to that of strain A23. “−” indicates that no sequence data were available in the DDBJ and EMBL/GenBank data libraries. ^a^The gene segments that are most similar to those of strain A14. ^b^The gene segments that are most similar to those of strain A23. ^c^The gene segments that are most similar to those of strain A25. ^d^The gene segments that are most similar to those of strain P02. ^e^The gene segments that are most similar to those of strain P39. ^f^The gene segments that are most similar to those of strain P43.

The 11 genes of strains A14, A23, A25, P02, P39, and P43 were assigned as G12-P[8]-I1-R1-C1-M1-A1-N1-T1-E1-H1 (strains A14, A25, P02, P39, and P43), and G12-P[6]-I1-R1-C1-M1-A1-N1-T1-E1-H1 (strain A23) (Fig 2). Strains A14, A23, A25, P02, P39, and P43 were confirmed to have the G12P[8], G12P[6], G12P[8], G12P[8], G12P[8], and G12P[8] genotypes, respectively, as determined by PCR-based genotyping (Ide et al., in preparation). Comparison of the complete genotype constellations of strains A14, A23, A25, P02, P39, and P43 with those of other G12 and non-G12 strains is shown in Fig 2. All the six Myanmarese G12 strains exhibited typical Wa-like genotype constellations, which are commonly found in the G12 strains recently detected worldwide (Rahman et al., 2007). Furthermore, as suggested by the genomic dsRNA profiles observed on PAGE analysis (Fig 1), strains A14, A25, P02, P39, and P43 exhibited extremely high nucleotide sequence identities (99.1–100%) to one another for all the 11 gene segments. On the other hand, the nucleotide sequence similarities of the VP4 and other 10 gene segments (VP7, VP6, VP1-3, and NSP1-5) of strain A23 to those of strains A14, A25, P02, P39, and P43 were comparatively low (75.3 and 93.1–98.7%, respectively).

### Phylogenetic analyses

We next constructed phylogenetic trees using the full-genome sequence for each of the 11 gene segments because phylogenetic analysis of RVA nucleotide sequences provides direct evidence of their relatedness to those of other strains, even within the same genotype [48].

**Fig 3:**
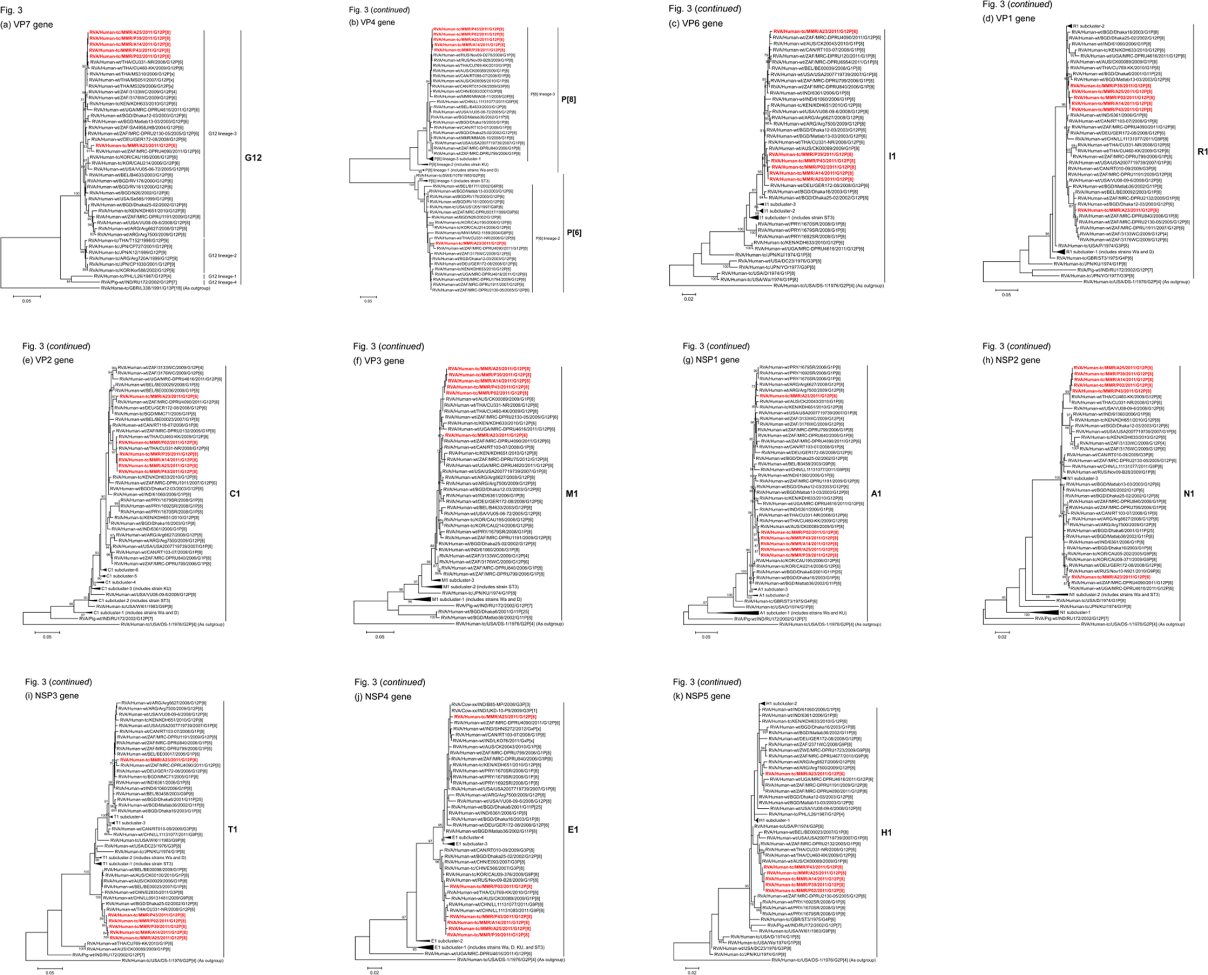
Phylogenetic tree constructed from the nucleotide sequences of the VP7 genes of strains A14, A23, A25, P02, P39, and P43, and representative RVA strains. In the tree, the positions of strains A14, A23, A25, P02, P39, and P43 are shown in red. Bootstrap values of <75% are not shown. Scale bars, 0.05 substitutions per nucleotide.

The VP7 genes of strains A14, A25, P02, P39, and P43 exhibited the maximum nucleotide sequence identities (99.7–99.8%) with that of Thai human strain CU331-NR (G12P[6]) [45] (Fig 2). On phylogenetic analysis, strains A14, A25, P02, P39, and P43 were found to form a cluster with strain CU331-NR and several human G12 strains from Thailand in G12 lineage-3, in which the majority of globally circulating G12 strains cluster [9, 10] (Fig 3). On the other hand, the VP7 gene of strain A23 showed the highest nucleotide sequence similarity (99.1%) with South African human strain MRC-DPRU4090 (G12P[6]) (Fig 2). Phylogenetically, strain A23 was found to be closely related with strain MRC-DPRU4090 in a common branch with German human strain GER172-08 (G12P[6]) [59] in G12 lineage-3 (Fig 3).

**Fig 4:**
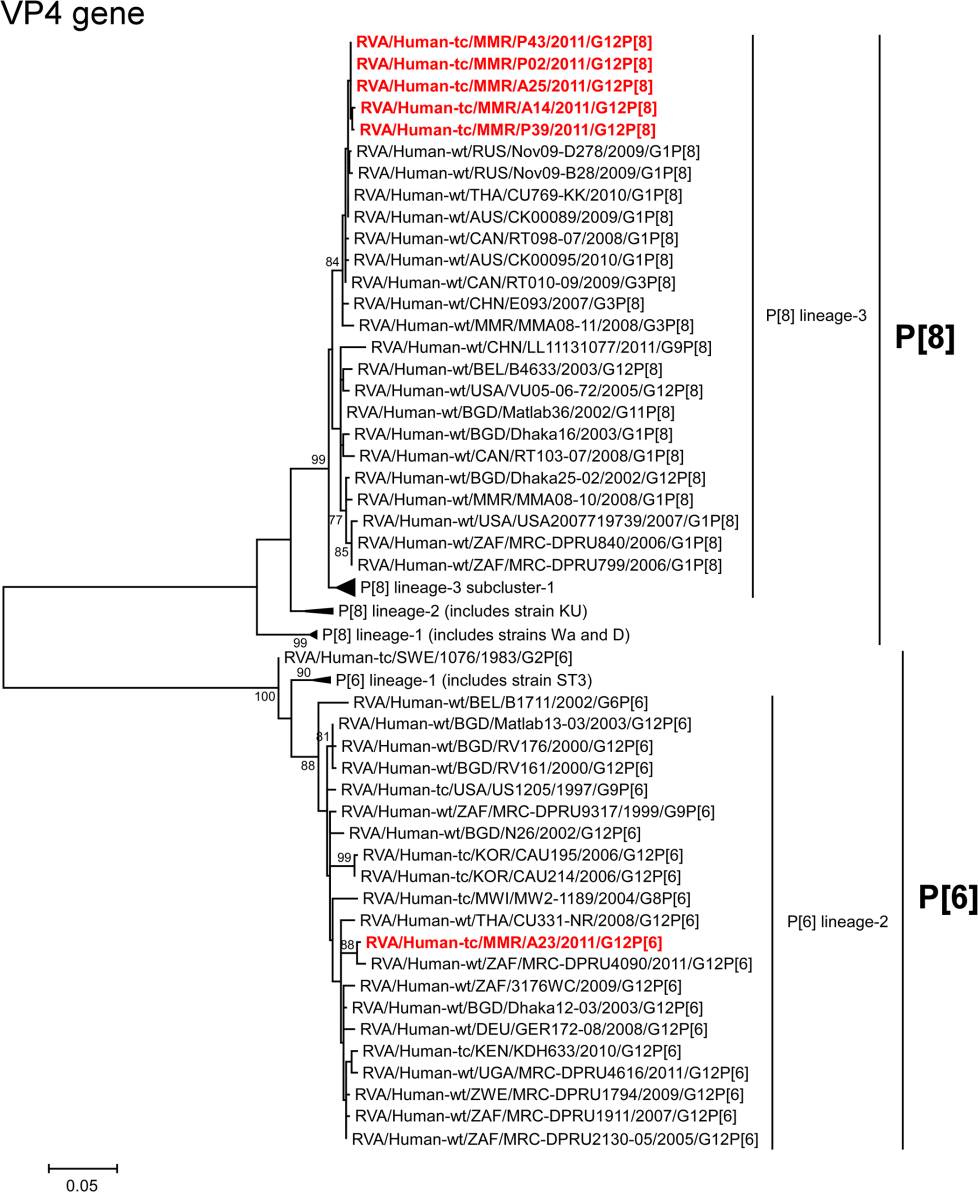
Phylogenetic tree constructed from the nucleotide sequences of the VP4 genes of strains A14, A23, A25, P02, P39, and P43, and representative RVA strains. See legend of Fig 3. Scale bars, 0.05 substitutions per nucleotide.

The VP4 genes of strains A14, A25, P02, P39, and P43 showed the highest nucleotide sequence identities (99.6%) with the cognate genes of Thai human strain CU769-KK (G1P[8]) [46], Russian human strain Nov09-B28 (G1P[8]), and/or Canadian human strain RT010-09 (G3P[8]) (Fig 2). On phylogenetic analysis, strains A14, A25, P02, P39, and P43 clustered near these, and several human G1 and G3 strains from different countries of the world in P[8] lineage-3 (Fig 4). In contrast, the VP4 gene of strain A23 exhibited the maximum nucleotide sequence identity (99.2%) with South African human strain MRC-DPRU4090 (G12P[6]) (Fig 2). Phylogenetically, strain A23 was found to be closely related with strain MRC-DPRU4090 in a common branch with several human G12 strains from different parts of the world in P[6] lineage-2 (Fig 4).

**Fig 5:**
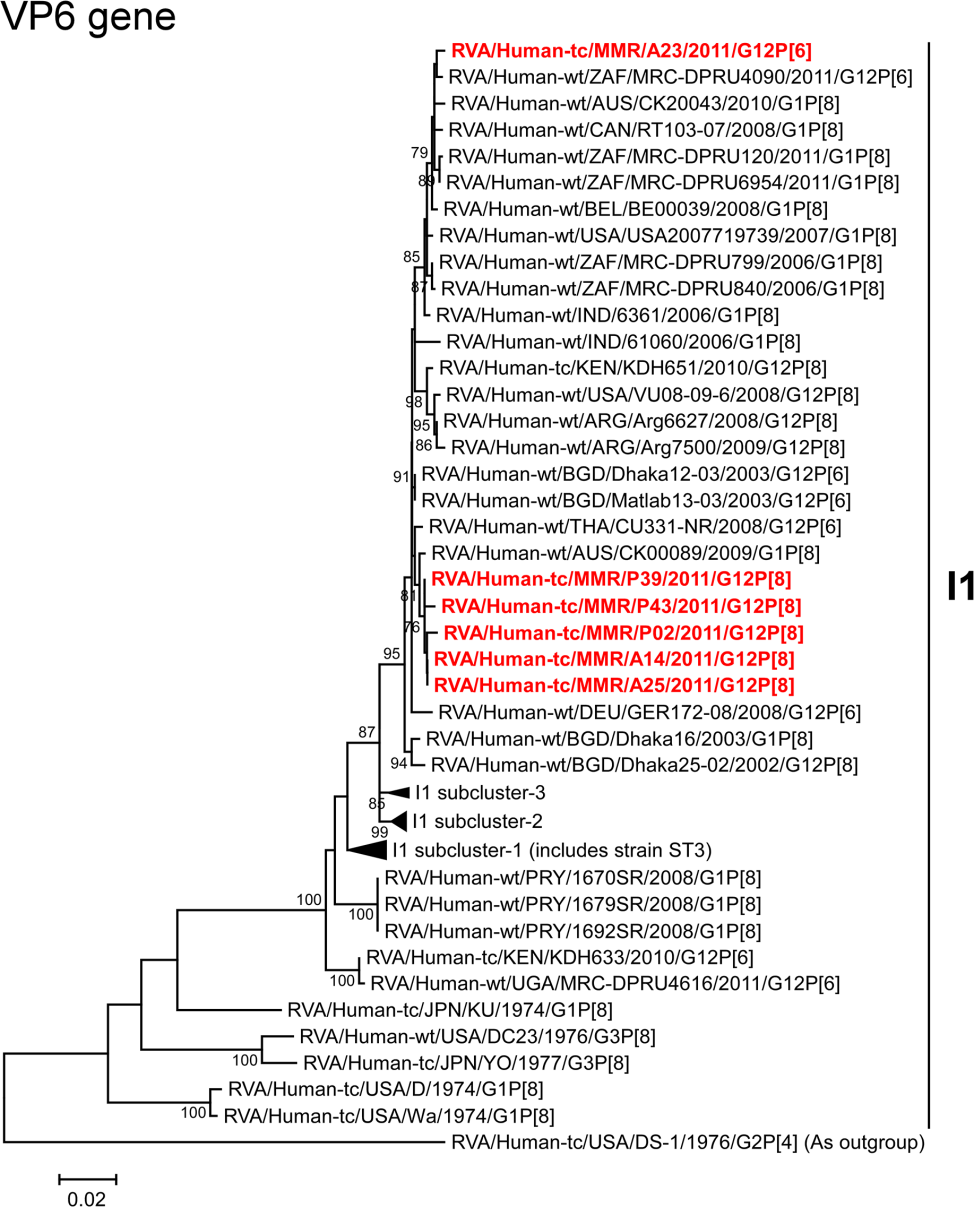
Phylogenetic tree constructed from the nucleotide sequences of the VP6 genes of strains A14, A23, A25, P02, P39, and P43, and representative RVA strains. See legend of Fig 3. Scale bars, 0.02 substitutions per nucleotide.

The VP6 genes of strains A14, A25, P02, P39, and P43 exhibited the highest nucleotide sequence identities (99.3–99.6%) with the VP6 gene of Australian human strain CK00089 (G1P[8]) (Fig 2). On phylogenetic analysis, strains A14, A25, P02, P39, and P43 were found to be closely related with strain CK00089 near several human G12 strains (Fig 5). On the other hand, the VP6 gene of strain A23 showed the maximum nucleotide similarity (99.6%) with South African human strain MRC-DPRU4090 (G12P[6]) (Fig 2). Phylogenetically, strain A23 was found to be clustered with strain MRC-DPRU4090 and several human G1 strains (Fig 5).

**Fig 6:**
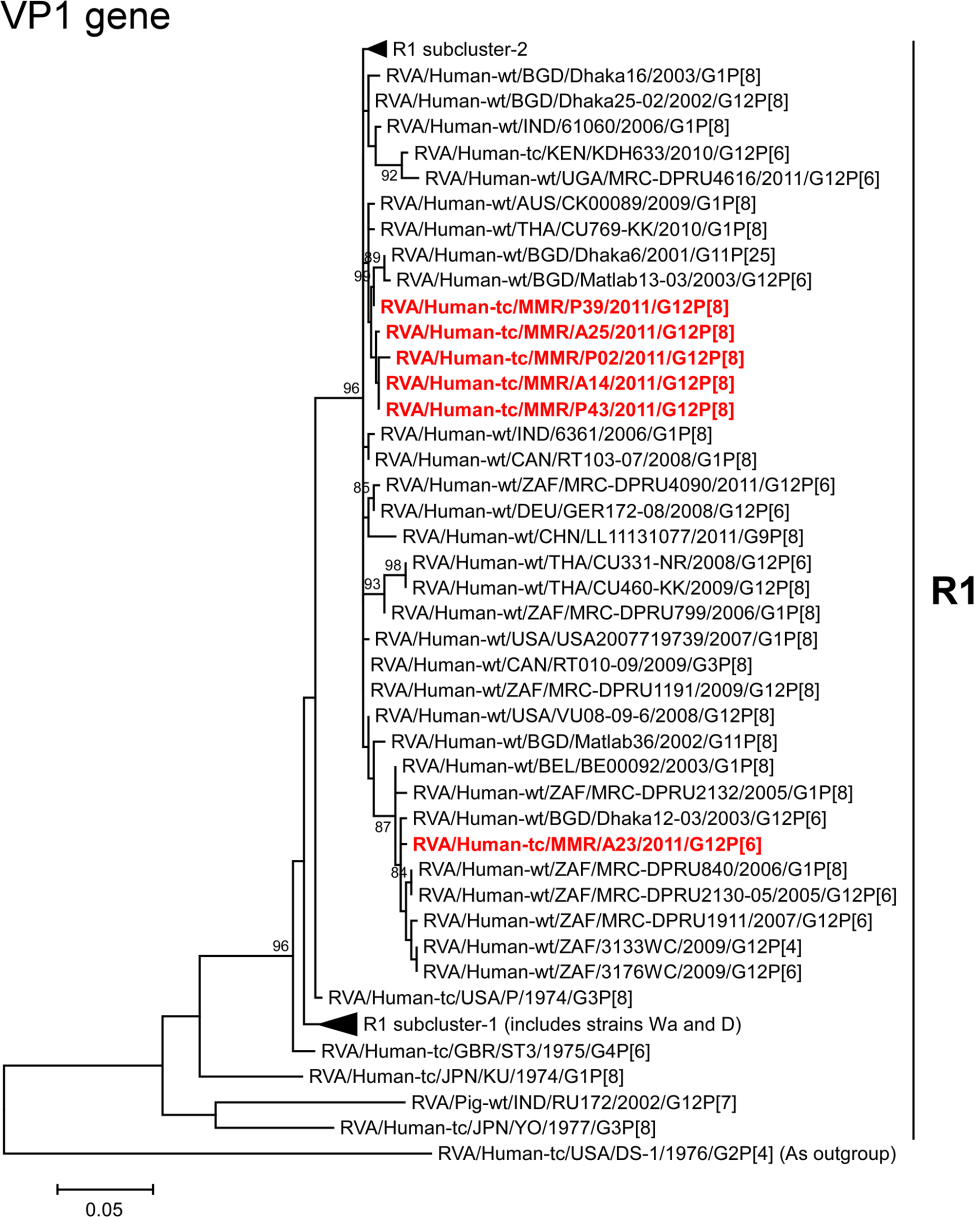
Phylogenetic tree constructed from the nucleotide sequences of the VP1 genes of strains A14, A23, A25, P02, P39, and P43, and representative RVA strains. See legend of Fig 3. Scale bars, 0.05 substitutions per nucleotide.

The VP1 genes of strains A14, A25, P02, P39, and P43 showed the maximum nucleotide sequence identities (99.6–99.7%) with the cognate gene of Australian human strain CK00089 (G1P[8]) (Fig 2). On phylogenetic analysis, strains A14, A25, P02, P39, and P43 were found to be clustered near strain CK00089, and several human G1, G11, and G12 strains (Fig 6). On the other hand, the VP1 gene of strain A23 exhibited the highest nucleotide sequence similarity (99.1%) with South African human strains MRC-DPRU2130-05 (G12P[6]) and MRC-DPRU840 (G1P[8]) (Fig 2). Phylogenetically, strain A23 clustered near these, and several human G1 and G12 strains (Fig 6).

**Fig 7:**
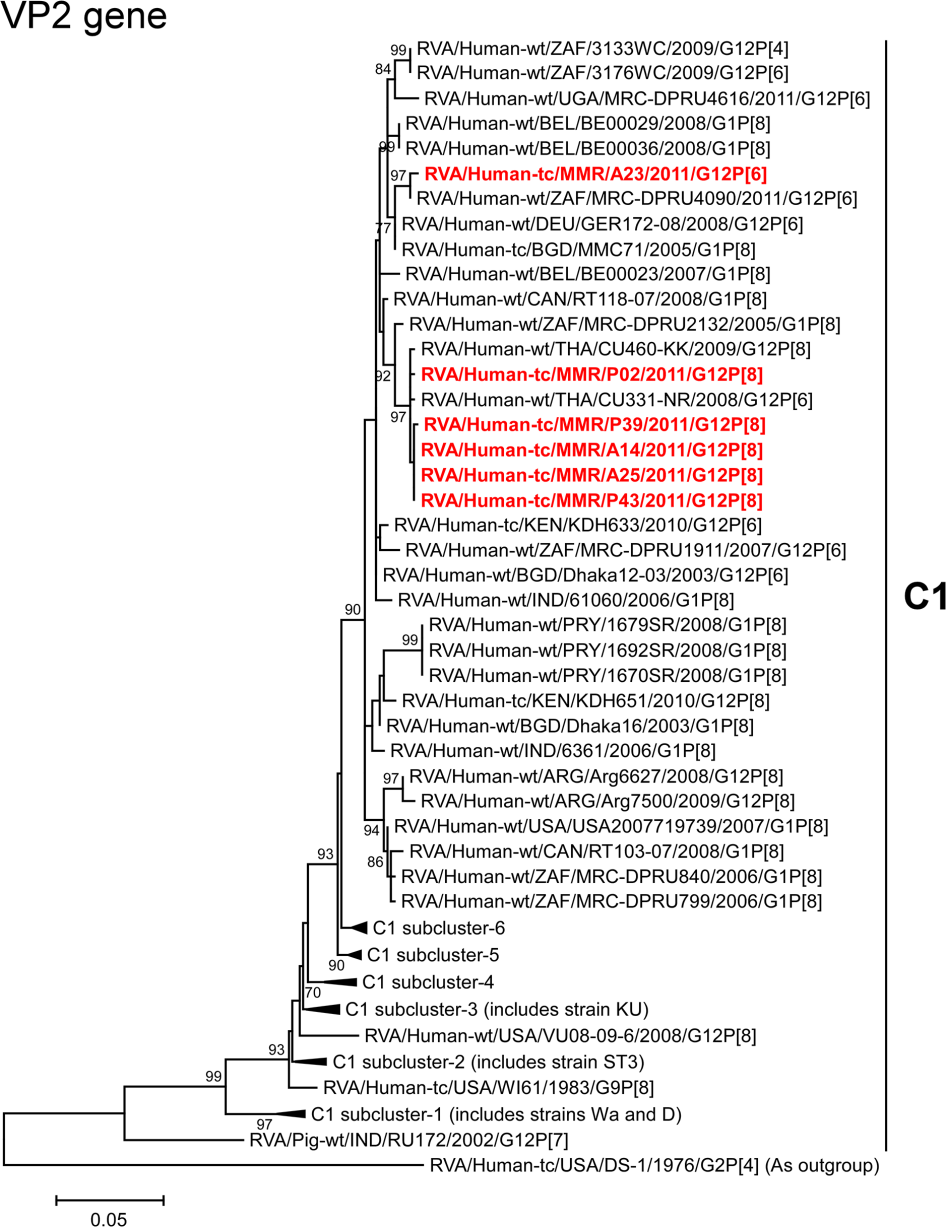
Phylogenetic tree constructed from the nucleotide sequences of the VP2 genes of strains A14, A23, A25, P02, P39, and P43, and representative RVA strains. See legend of Fig 3. Scale bars, 0.05 substitutions per nucleotide.

The VP2 genes of strains A14, A25, P02, P39, and P43 showed the highest nucleotide sequence similarities (99.6–99.7%) with the VP2 genes of Thai human strains CU331-NR (G12P[6]) and/or CU460-KK (G12P[8]) [45] (Fig 2). On phylogenetic analysis, strains A14, A25, P02, P39, and P43 were found to be closely related with these strains in a common branch with South African human strain MRC-DPRU2132 (G1P[8]) (Fig 7). In contrast, the VP2 gene of strain A23 exhibited the maximum nucleotide sequence identity (99.4%) with South African human strain MRC-DPRU4090 (G12P[6]) (Fig 2). On phylogenetic analysis, strain A23 was found to be closely related with strain MRC-DPRU4090 in a common branch with German human strain GER172-08 (G12P[6]) and Bangladeshi human strain MMC71 (G1P[8]) [60] (Fig 7).

**Fig 8:**
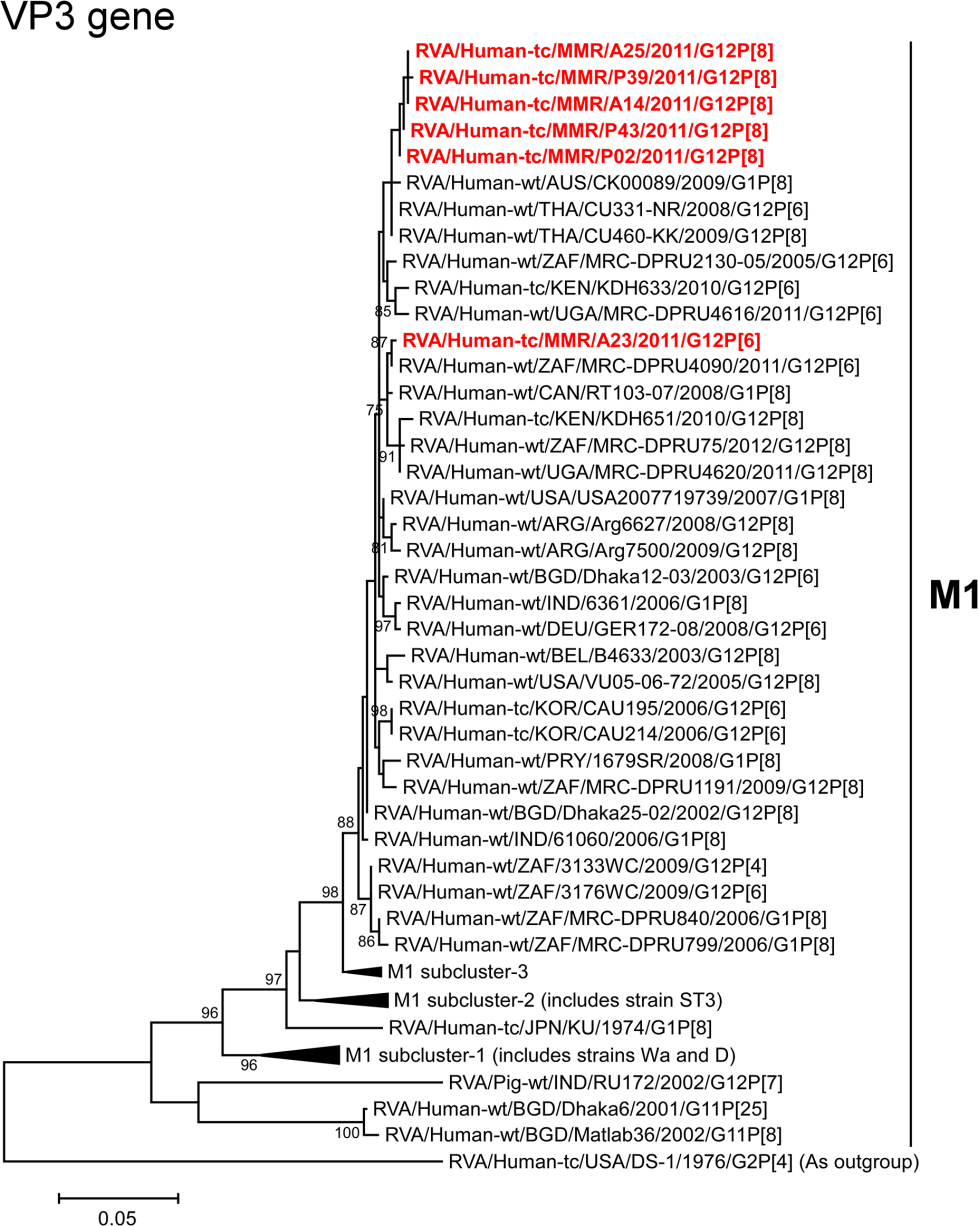
Phylogenetic tree constructed from the nucleotide sequences of the VP3 genes of strains A14, A23, A25, P02, P39, and P43, and representative RVA strains. See legend of Fig 3. Scale bars, 0.05 substitutions per nucleotide.

The VP3 genes of strains A14, A25, P02, P39, and P43 showed the maximum nucleotide sequence identities (99.7–99.8%) with those of Thai human strains CU331-NR (G12P[6]) and/or CU460-KK (G12P[8]) (Fig 2). On phylogenetic analysis, strains A14, A25, P02, P39, and P43 were found to be clustered with these strains, and several human G1 and G12 strains (Fig 8). On the other hand, the VP3 gene of strain A23 exhibited the highest nucleotide sequence similarity (99.5%) with Canadian human strain RT103-07 (G1P[8]) (Fig 2). Phylogenetically, strain A23 was found to be closely related with South African human strain MRC-DPRU4090 (G12P[6]) in a common branch with strain RT103-07 and several human G12 strains from Africa (Fig 8).

**Fig 9:**
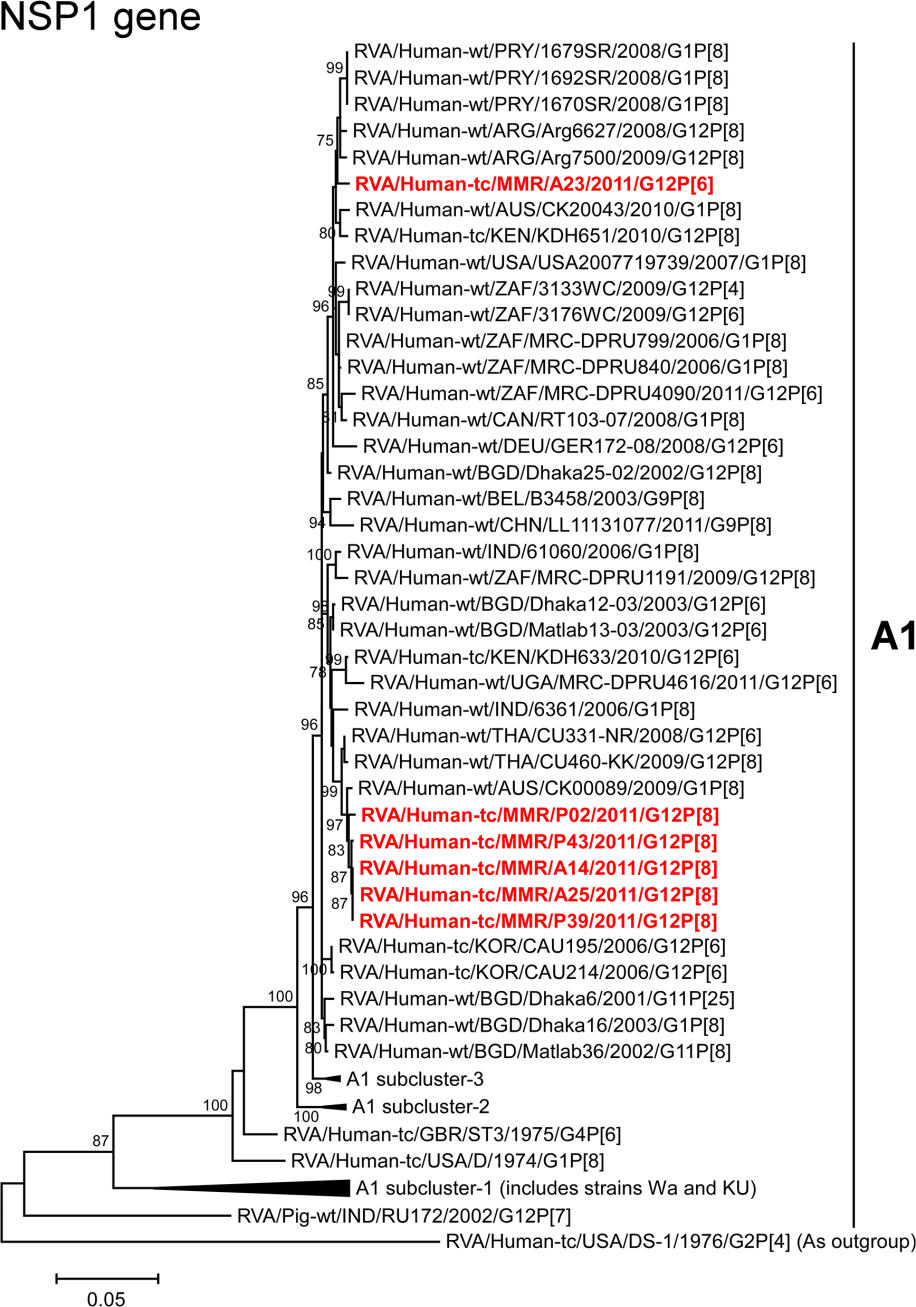
Phylogenetic tree constructed from the nucleotide sequences of the NSP1 genes of strains A14, A23, A25, P02, P39, and P43, and representative RVA strains. See legend of Fig 3. Scale bars, 0.05 substitutions per nucleotide.

The NSP1 genes of strains A14, A25, P02, P39, and P43 exhibited the highest nucleotide sequence identities (99.5–99.6%) with the cognate gene of Australian human strain CK00089 (G1P[8]) (Fig 2). On phylogenetic analysis, strains A14, A25, P02, P39, and P43 were found to be closely related with strain CK00089 in a common branch with Thai human strains CU331-NR (G12P[6]) and CU460-KK (G12P[8]) (Fig 9). On the other hand, the NSP1 gene of strain A23 showed the maximum nucleotide sequence similarity (99.0%) with Argentinian human strains (Arg6627 (G12P[8]) and Arg7500 (G12P[8]) [61]), Paraguayan human strains (1670SR (G1P[8]), 1679SR (G1P[8]), and 1692SR (G1P[8])), and South African human strain MRC-DPRU799 (G1P[8]) (Fig 2). Phylogenetically, strain A23 was found to be clustered near these strains, and several human G1 and G12 strains (Fig 9).

**Fig 10:**
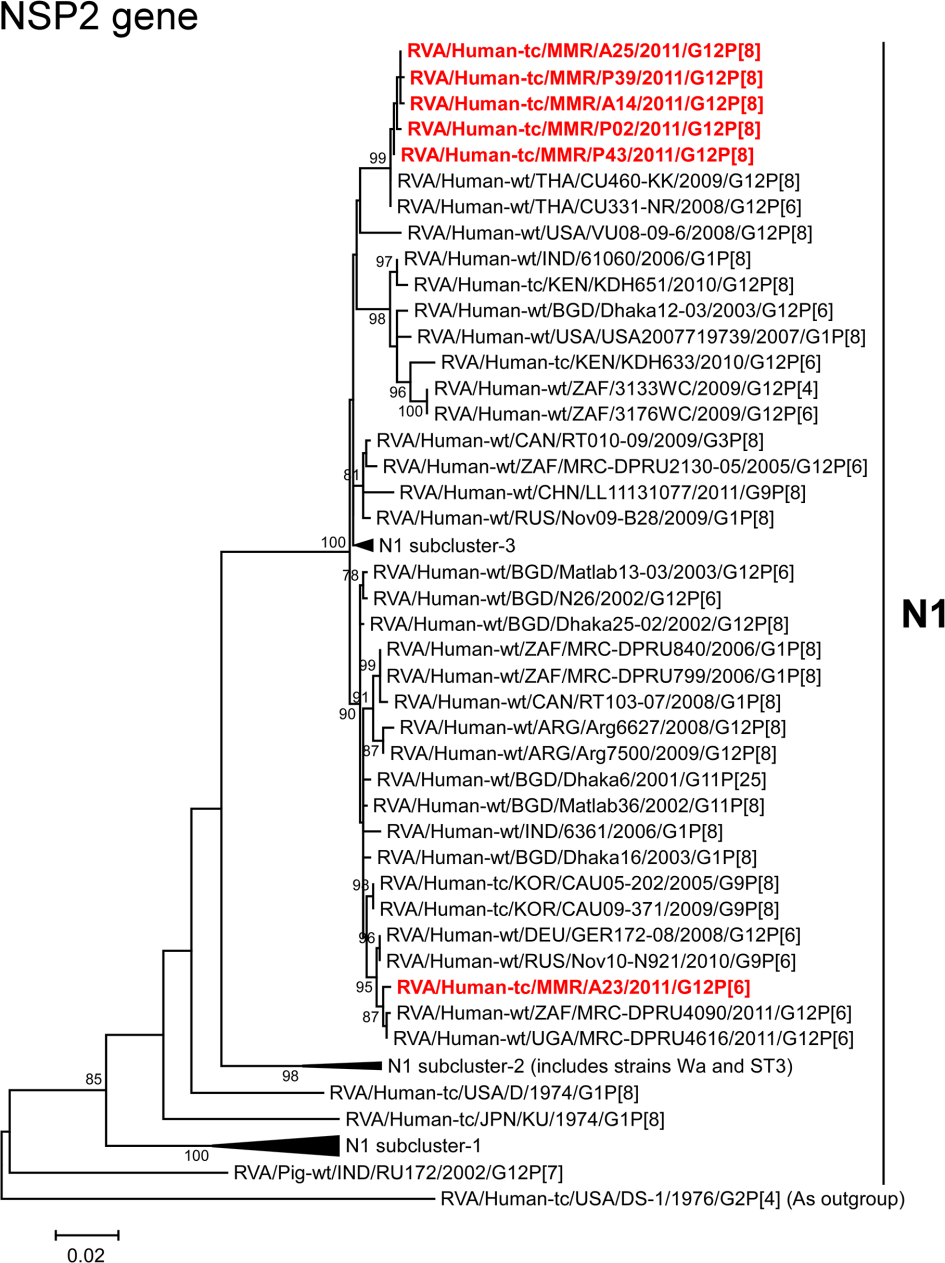
Phylogenetic tree constructed from the nucleotide sequences of the NSP2 genes of strains A14, A23, A25, P02, P39, and P43, and representative RVA strains. See legend of Fig 3. Scale bars, 0.02 substitutions per nucleotide.

The NSP2 genes of strains A14, A25, P02, P39, and P43 exhibited the maximum nucleotide sequence identities (99.6–99.8%) with those of Thai human strains CU331-NR (G12P[6]) and CU460-KK (G12P[8]) (Fig 2). On phylogenetic analysis, strains A14, A25, P02, P39, and P43 were found to be closely related with these strains (Fig 10). On the other hand, the NSP2 gene of strain A23 showed the highest nucleotide sequence identity (99.8%) with Ugandan human strain MRC-DPRU4616 (G12P[6]), and comparable identity (99.7%) with South African human strain MRC-DPRU4090 (G12P[6]) (Fig 2). Phylogenetically, strain A23 was found to be closely related with these strains in a common branch with German human strain GER172-08 (G12P[6]) and Russian human strain Nov10-N921 (G9P[6]) [62] (Fig 10).

**Fig 11:**
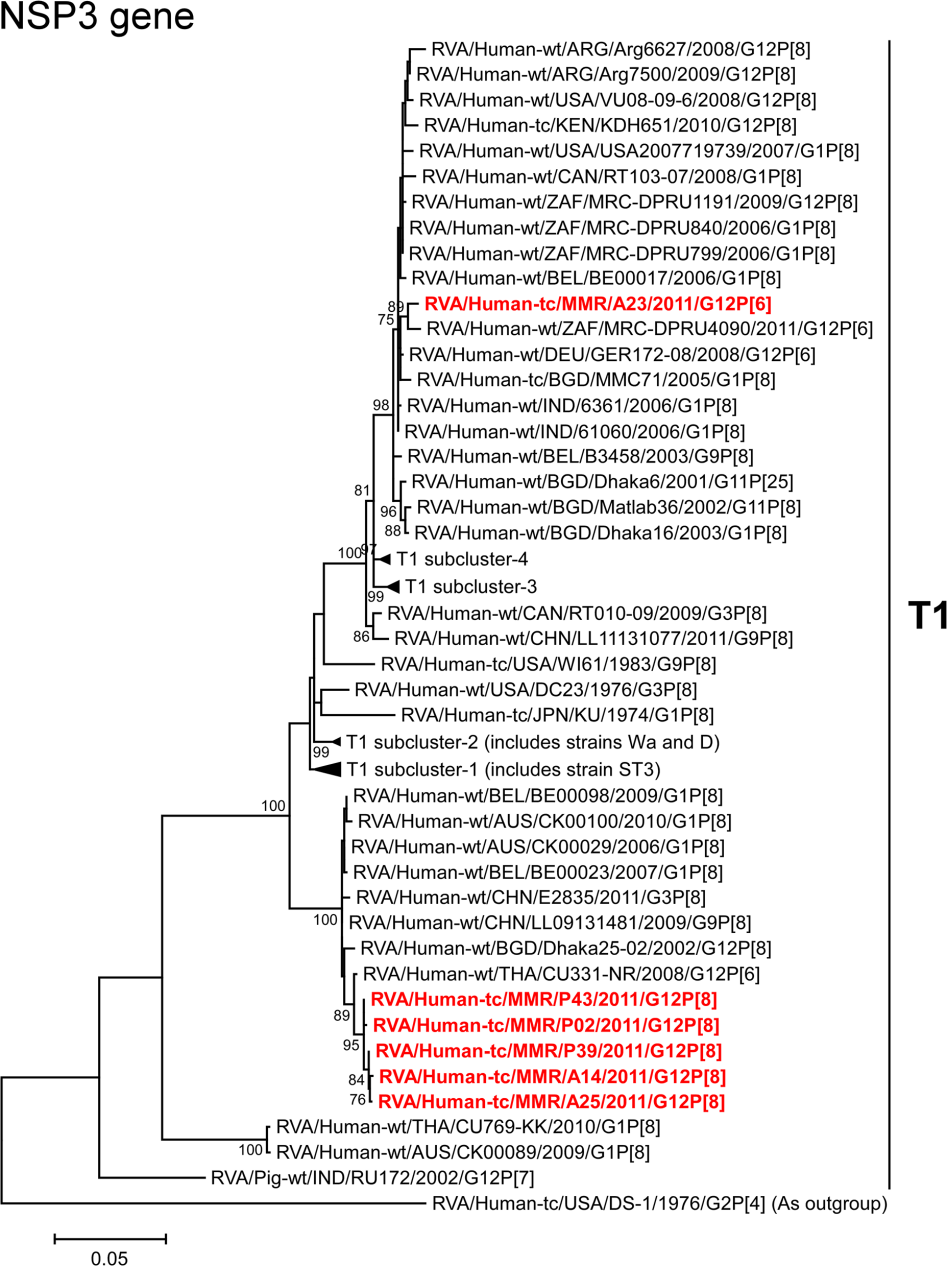
Phylogenetic tree constructed from the nucleotide sequences of the NSP3 genes of strains A14, A23, A25, P02, P39, and P43, and representative RVA strains. See legend of Fig 3. Scale bars, 0.05 substitutions per nucleotide.

The NSP3 genes of strains A14, A25, P02, P39, and P43 showed the highest nucleotide sequence identities (99.3–99.5%) with the NSP3 gene of Thai human strain CU331-NR (G12P[6]) (Fig 2). On phylogenetic analysis, strains A14, A25, P02, P39, and P43 were found to be closely related with strain CU331-NR in a common branch with several human G1, G3, G9, and G12 strains (Fig 11). In contrast, the NSP3 gene of strain A23 exhibited the maximum nucleotide sequence identity (99.2%) with German human strain GER172-08 (G12P[6]), and comparable identity (99.1%) with South African human strains MRC-DPRU4090 (G12P[6]), MRC-DPRU799 (G1P[8]), and MRC-DPRU840 (G1P[8]) (Fig 2). On phylogenetic analysis, strain A23 was found to be closely related with strain MRC-DPRU4090 in a common branch with these, and several human G1 and G12 strains (Fig 11).

**Fig 12:**
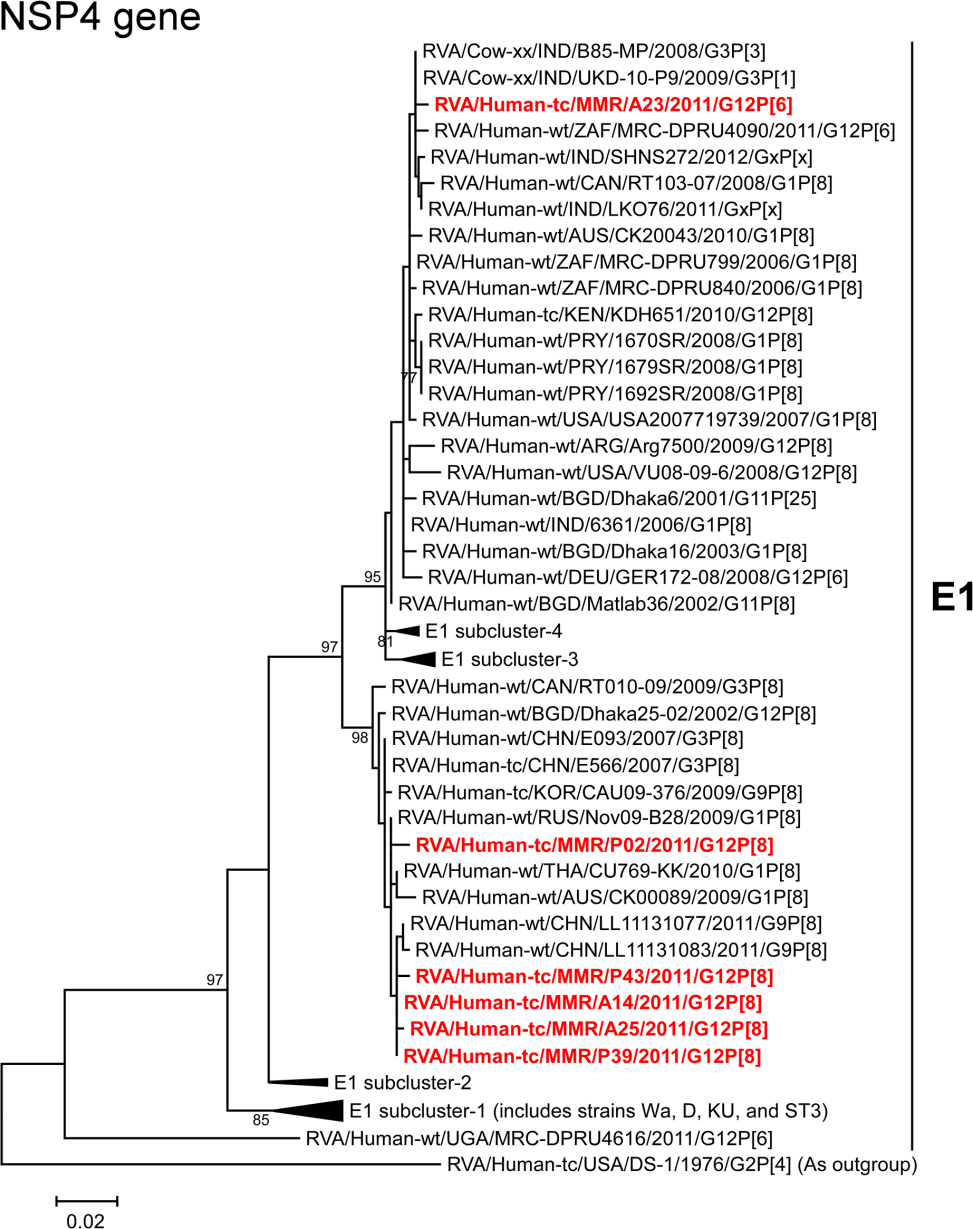
Phylogenetic tree constructed from the nucleotide sequences of the NSP4 genes of strains A14, A23, A25, P02, P39, and P43, and representative RVA strains. See legend of Fig 3. Scale bars, 0.02 substitutions per nucleotide.

The NSP4 genes of strains A14, A25, P02, P39, and P43 showed the maximum nucleotide sequence similarities (99.4–99.6%) with the cognate genes of Thai human strain CU769-KK (G1P[8]), Chinese human strain LL11131077 (G9P[8]), and/or Russian human strain Nov09-B28 (G1P[8]) (Fig 2). On phylogenetic analysis, strains A14, A25, P02, P39, and P43 were found to be clustered near these, and several human G1, G3, G9, and G12 strains (Fig 12). In contrast, the NSP4 gene of strain A23 exhibited the highest nucleotide sequence identity (99.5%) with Indian human-like bovine strain B85-MP (G3P[3]), South African human strain MRC-DPRU799 (G1P[8]), and American human strain USA2007719739 (G1P[8]) (Fig 2). Phylogenetically, A23 was found to be clustered with these, and several human-like bovine and human strains within the human-like E1 subcluster (Fig 12).

**Fig 13:**
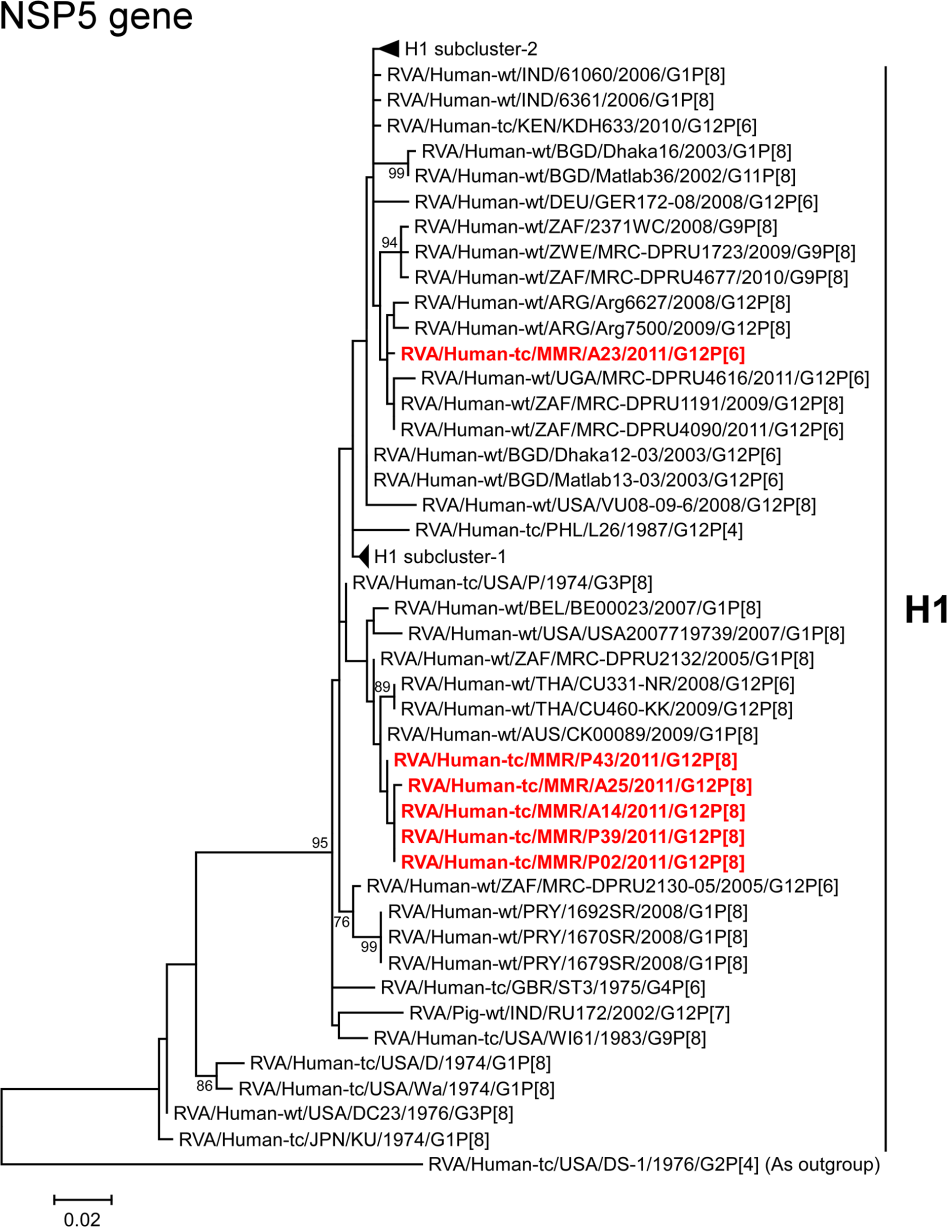
Phylogenetic tree constructed from the nucleotide sequences of the NSP5 genes of strains A14, A23, A25, P02, P39, and P43, and representative RVA strains. See legend of Fig 3. Scale bars, 0.02 substitutions per nucleotide.

The NSP5 genes of strains A14, A25, P02, P39, and P43 exhibited the maximum nucleotide sequence identities (99.6–99.8%) with that of Australian human strain CK00089 (G1P[8]) (Fig 2). On phylogenetic analysis, strains A14, A25, P02, P39, and P43 were found to be clustered with strain CK00089, and several human G1 and G12 strains (Fig 13). On the other hand, the NSP5 gene of strain A23 showed the maximum nucleotide sequence similarity (99.6%) with South African human strains MRC-DPRU4090 (G12P[6]) and MRC-DPRU1191 (G12P[8]) (Fig 2). On phylogenetic analysis, strain A23 was found to be clustered near these, and several human G9 and G12 strains (Fig 13).

In the present study, we analyzed the whole genomes of six Asian G12 strains that have emerged in Myanmar (strains A14, A23, A25, P02, P39, and P43) identified in six stool specimens from children with gastroenteritis. All six Myanmarese G12 strains were found to have a Wa-like backbone: G12-P[8]-I1-R1-C1-M1-A1-N1-T1-E1-H1 (strains A14, A25, P02, P39, and P43) and G12-P[6]-I1-R1-C1-M1-A1-N1-T1-E1-H1 (strain A23). Whole genomic analyses of currently circulating G12 strains from different parts of the world have revealed the predominant possession of a Wa-like genetic backbone by them [10]. Because a Wa-like genotype backbone is believed to be one of the stable genetic constellations of human RVAs, the Wa-like backbone might have conferred an evolutionary advantage on G12 strains, making it possible for them to spread across the globe in an incredibly short time period [48, 50, 63]. Phylogenetic analysis revealed that in most genes the six strains examined in this study were genetically related to globally circulating human G1, G3, G9, and G12 strains. Furthermore, strains A14, A25, P02, P39, and P43 were very closely related to one another as to all the 11 genes, indicating the derivation of the five strains from a common origin. On the other hand, strain A23 consistently formed distinct clusters for all the 11 gene segments, indicating its distinct origin from that of strains A14, A25, P02, P39, and P43. While eight of the 11 gene segments (VP7, VP4, VP2-3, and NSP1-4) of strains A14, A25, P02, P39, and P43 were very closely related to those of Asian strains, all the 11 gene segments of strain A23 were very closely associated with African strains. Thus, the G12 strains that have emerged in Myanmar appeared to have originated from not a single, but at least two distinct ancestral G12 strains.

Notably, the NSP4 gene of strain A23 having E1 genotype showed the closest relationship with the NSP4 genes of human-like bovine strains from India, as well as human strains (Fig 12). Genotype E1 has been recently detected in bovines in India and Korea, in addition to more common bovine genotype E2 [64, 65]. However, the exact origin of the NSP4 gene of strain A23 could not be ascertained due to a lack of a sufficient number of representative strains as references. In any case, this observation suggests the occurrence of reassortment between human and bovine strains. To our knowledge, this is the first description on full genome-based characterization of the G12 strains that have emerged in Myanmar.

The increasing detection of G12 strains in Asia implies the continued spread of G12 strains in Asia. Because it has not been examined as to whether or not the two available RVA vaccines (Rotarix (GlaxoSmithKline) and RotaTeq (Merck)) are effective for prevention against unusual strains, such as G12P[6] strains that share no G/P genotype specificities with strains included in these vaccines, continuing RVA surveillance of the G12 genotype may be required. Simultaneous monitoring of RVA strains in humans and animals is also essential for a better understanding of RVA ecology. Furthermore, whole genome-based analyses are essential to understand the evolutionary dynamics of G12 strains spreading in Asia.
